# In Vitro and In Vivo Study of Combined Effect of Some Algerian Medicinal Plants and Probiotics against *Helicobacter pylori*

**DOI:** 10.3390/microorganisms11051242

**Published:** 2023-05-08

**Authors:** Bouhenni Hasna, Hemida Houari, Doukani Koula, Spinu Marina, Ungureanu Emilia, Boumezrag Assia

**Affiliations:** 1 Faculty of Nature and Life Sciences, University of Tiaret, Tiaret 14000, Algeria; 2 Institute of Veterinary Sciences, University of Tiaret, Tiaret 14000, Algeria; 3 Department of Infectious Diseases, Clinical Sciences, University of Agricultural Sciences and Veterinary Medicine, 400372 Cluj-Napoca, Romania

**Keywords:** *Helicobacter pylori*, probiotics, fenugreek, cumin, garlic, onion, in vivo, combined effect

## Abstract

*Helicobacter pylori* causes gastritis, peptic ulcers and gastric cancer and affects more than half of the world’s population. Although this infection can have serious consequences, no novel cure or remedy have been discovered, the present therapy still relies on a variety of known antibiotics and anti-secretory agents. In the present study, the potential effect of combinations between methanolic extracts of four Algerian medicinal plants, garlic (*Allium sativum*), red onion (*Allium cepa*), cumin (*Cuminum cyminum* L.) and fenugreek (*T. foenum-graecum* L.), with different strains of lactic acid bacteria against *H. pylori*, was investigated. Similarly, the in vivo antibacterial effect of fenugreek extract combined with *Bifidobacterium breve* on *H. pylori* colonization potential to confirm the enhanced effect of the mixture was explored. *Helicobacter pylori* was inhibited by all combined mixtures of extracts and probiotics with varying results. The highest anti-*H. pylori* activities were found in fenugreek/*B. breve*, cumin/*B. breve*, garlic/*B. breve* and onion/*B. breve* combinations with inhibition diameters of 29, 26, 23 and 25 mm, respectively. Preliminary studies on the effect of probiotics against *H. pylori* revealed that the inhibition was due to lactic acid and bacteriocins and it was also related to the presence of phenolic compounds in the studied plants, such as gallic acid, caffeic acid, quercetin and vanillic acid. Fenugreek extract was found to inhibit the growth of H. pylori in a concentration-dependent manner. When *H. pylori*-infected rats were administered *B. breve*, the infection rate of *H. pylori* was significantly reduced, while the combination of *B. breve* and fenugreek extract effectively inhibited *H. pylori*. In addition, the B. breve and fenugreek extract mixture significantly reduced gastritis in *H. pylori*-infected rats. These results suggest that this complex mixture may be an alternative to treating diseases caused by *H. pylori* infection.

## 1. Introduction

*Helicobacter pylori* (*H. pylori*) is a Gram-negative spiral-shaped bacterium, which causes gastric infection, leading to diverse outcomes ranging from asymptomatic gastritis to invasive adenocarcinoma [1,2]. Numerous factors have been identified that may contribute to the development of adverse *H. pylori* infection outcomes; these can be grouped into a triad of risk factors, including host, pathogen and environmental characteristics that interact to promote disease progression [2]. Over half of the people on the planet are its hosts, making this bacterium one of the most common infectious factors in humans [3]. About 70% of the patients showing *H. pylori* infection of the stomach display no symptoms, only around 10% to 20% will likely develop peptic ulcers and only 1–2 percent will be diagnosed with gastric cancer or mucosa-associated lymphoid tissue lymphoma [4]. In a previous study in Algeria, the prevalence of H. pylori infection was 66.1%, more prevalent in the 60–69 age group (71.43%) and statistically higher in women than in men (69.3% vs. 60.7%) [5]. Raaf et al., (2017) reported that the prevalence of *H. pylori* infection remains high in Algeria but appears to have decreased in recent years [6]. The decrease in the prevalence of this infection requires the improvement of sanitary conditions and novel therapeutic strategies in place for those patients suffering from *H. pylori*-related gastric diseases [5]. The classical treatment to eradicate this bacterium from humans involves the use of therapies that include antibiotics, such as clarithromycin, metronidazole or levofloxacin, together with a proton pump inhibitor for a duration of 10 to 14 days [7]. The primary method of treating this infection is the use of antibiotics; however, due to the length of the therapy and the subsequent secondary effects induced by this, alternative methods of treatment should be sought and investigated [8]. According to the World Health Organization (2017), *H. pylori* that is resistant to clarithromycin falls under the high-priority category in antimicrobial research development and has to be treated as directed [9]. Such a conduct has been conditioned by a sharply growing drug resistance and, as a result, loss of effectiveness of therapy regimens using clarithromycin for the treatment of *H. pylori* infection [10,11]. The bismuth quadruple therapy consists of two antibiotics, tetracycline and metronidazole, plus bismuth and a Proton pump inhibitor (PPI) for a period of 14 days [5]. This therapy is preferred as a first-line treatment option in areas with a high prevalence of clarithromycin resistance and also as a second-line therapy when initial treatment with the classic triple therapy for *H. pylori* has failed [7].

Consequently, a lot of research has focused on treating *H. pylori* infections using herbal remedies. There have been reports of anti-*H. pylori* activity in a variety of herbal remedies that have historically been used to treat cutaneous wounds and digestive problems [12]. Bi et al., (2014) [13] showed that herbal remedies are efficient in healing stomach ulcers, have fewer adverse effects and have reduced recurrence rates when compared to conventional drugs [13]. Additionally, combining herbal remedies with traditional anti-gastric ulcer medications has a synergistic impact on stomach ulcers [14]. Since most plants contain medicinal chemicals, they can be utilized to treat a variety of ailments [15,16]. Until recently, researchers have evaluated the antibacterial effects of a number of herbal extracts against *H. pylori*, including those from garlic, onions, cumin and fenugreek, and their results have indicated that some herbs are able to inhibit the growth of *H. pylori* [17,18,19,20].

Probiotic bacteria are described as living microorganisms that, when given to a host in sufficient quantities, improve their health. Probiotic activity has been associated with *Bifidobacteria*, *Lactobacilli*, *Streptococcus*, *Enterococcus* and *Saccharomyces boulardii* [21]. In order to effectively combat infections, probiotic bacteria must not be pathogenic and must generate antibacterial compounds [22]. In order to colonize the gastrointestinal epithelium and persist in situ, they also need to be tolerant to bile salts and gastric acid [23]. Characteristics considered important for probiotic efficacy could be indicative of possible mechanisms that underlie the observed clinical findings. Such characterizations could include survival at relevant body sites, the production of lactic acid or other short-chain fatty acids, adhesion to mucus or intestinal epithelial cells, interaction with human immune cells, resistance to digestive enzymes, bile or acid, antibacterial activity via competitive exclusion or production of bacteriocins or hydrogen peroxide [24]. Probiotics have been proven effective by acting through the competitive exclusion of the pathogenic bacteria [25] from the gastrointestinal tract. It is well established that lactobacilli (*L. acidophilus* R0052, *L. rhamnosus* R0011, *L. casei* Shirota strain and *L. johnsonii* strain La1) may kill *H. pylori* in vitro and, to a lesser extent, in vivo [26,27]. Lactobacilli-containing probiotics as an adjunct had a positive effect on improving eradication rates and reducing therapy-related side effects [28]. Furthermore, *Lactobacillus* administered alone is shown to significantly improve eradicating therapy [29]. Recent research has shown that using oral probiotics is quite helpful for treating *H. pylori* infection [30]. Natural medicines and probiotics could prove to be a useful alternative to pharmaceuticals for treating the infection since they appear to have an increased efficiency with minimal side effects [22].

The possibility of combined therapy using phenolic phytochemical-linked medicinal plants and lactic acid bacteria with probiotic potential on *H. pylori* inhibition may represent a novel and affordable approach to treating *H. pylori*-associated peptic ulcers and active chronic gastritis and impacting their long-term effects [31].

Based on the rationale above, the objective of this research was to evaluate the antimicrobial potential of combined methanolic extracts of some medicinal plants from Algeria, garlic, onion, cumin and fenugreek, with different probiotic strains, such as *Bifidobacterium breve*, *Bifidobacterium bifidum*, *Bifidobacterium longum*, *Lacticaseibacillus rhamnosus GG*, *Lacticaseibacillus rhamnosus LA80*, *Lactobacillus helviticus*, *Limosilactobacillus fermentum*, *Lactiplantibacillus plantarum*, *Lacticaseibacillus casei*, *Lactobaccilus acidophilus*, *Lactobacillus lactis* and *Streptococcus thermophilus,* against *H. pylori* both in vitro and in vivo. To our best knowledge, this study is the first report on the effect of combined Algerian medicinal plants with probiotics against *H. pylori* responsible for gastro-duodenal diseases.

## 2. Material and Methods

### 2.1. Materials

#### 2.1.1. Plant Material

Samples of garlic (*Allium sativum*) bulb, onion (*Allium cepa*) bulb, fenugreek (*Trigonella foenum-graecum* L.) seeds and cumin (*Cuminum cyminum* L.) seeds were sorted, washed and dried at room temperature, then ground into a fine powder and sieved through a 200 μm sieve. Resulting powders were used to prepare methanolic extracts using a maceration method; all the extraction procedures were performed and conditions were set as described by Bouhenni et al., (2019_a_) and Bouhenni et al., (2019_b_) [32,33]. Briefly, the methods applied to investigate the chemical composition of the tested plant extracts were:

##### Determination of Total Phenolic Content

An amount of 0.5 mL of varying concentrations of each used extract and 2.5 mL of Folin–Ciocalteu (1/10 dilution in water) were mixed with 1 mL of sodium carbonate (20%). This mixture was incubated in the dark at room temperature for 30 min. The absorbance of the solution was measured at 765 nm using a UV Vis spectrophotometer A calibration curve was established using gallic acid as standard. The results were expressed as milligrams of gallic acid equivalent (GAE) per 100 g of Dry Matter.

##### Determination of Total Flavonoids Content

The total flavonoid content of plant extracts was determined using the aluminium chloride method. 1.5 mL of various concentrations was mixed with 75 µL of aluminium chloride solution and 0.5 mL of sodium acetate solution; the mixture was completed with distilled water until a volume of 2.5 mL. After an incubation period of 30 min at room temperature in the dark, the absorbance of the solution was measured at 415 nm using a UV-Vis spectrophotometer. The results were expressed as milligrams of quercitin equivalent (QE) per 100 g of Dry Matter.

##### Determination of Condensed Tannins Content

An amount of 1 mL of each extract was mixed with 2.5 mL of 4% methanol vanillin solution and 2.5 mL of H_2_SO_4_. After 15 min, the absorbance was measured at 500 nm. Condensed tannin contents were expressed as milligrams of Catechin equivalent (CE) per 100 g of Dry Matter.

##### Determination of Hydrolysable Tannins Content

An amount of 500 µL of the extract was added to 3.5 mL of the ferric chloride solution. The contents were then quickly mixed and the absorbance read at 660 nm, 15 secs after the addition of the extract solution. Hydrolysable tannin content was expressed as milligrams of Tannic acid equivalent (TAE) per 100 g of Dry Matter. 

Phenolic compounds were identified qualitatively by the HPLC method (Supplementary Materials) and antioxidant activity was measured using a DPPH assay [32,33]. The extracts were carefully stored and later used for assessing their anti-*H. pylori* effects.

#### 2.1.2. Probiotic Strains

Twelve probiotic strains were used in this study (Table 1): six of them (6) were obtained from the culture collection of the Laboratory of Sciences and Technics of Animal Production, Faculty of Agriculture-Abdelhamid Ibn Badis University, Mostaganem-Algeria; one (1) strain was obtained from the Laboratory of Natural and Local Bioresources, University of Hassiba Benbouali, Chlef-Algeria and the remaining strains (5) were purchased as commercial products in dried form: *Lacticaseibacillus rhamnosus* GG (Probiolog^®^ Laboratoires Mayoly Spindler, Chatou, France), *Lacticaseibacillus rhamnosus* LA80 (LACTIBIANE^®^ laboratoire PileJe, Orée-d’Anjou, France), *Lactobacillus helviticus* (Laxatransit^®^, laboratoire 3C Pharma, La Chapelle-d’Angillon, France), *Bifidobacterium bifidum* and *Bifidobacterium longum* (BENEFLORA^®^ and Laboratoires ORTIS, Bütgenbach, Belgium) [34].

#### 2.1.3. *H. pylori Strains*

Fresh gastric biopsy specimens obtained from patients who underwent upper gastrointestinal endoscopy or from symptomatic patients with a positive rapid urease assay at the Youcef Damardji Tiaret Hospital, Algeria were transferred to sterile physiological saline, transported to the laboratory and processed within 4 h after sampling. Biopsy specimens were minced and homogenized in physiological sterile saline (0.5 mL) and then plated immediately on plates containing Colombia agar supplemented with 5% horse blood. The plates were incubated at 37 °C under microaerophilic conditions (10% CO_2_) using BD GasPak™ (kingtec, Taiwan) for a maximum of 10 days. Suspected isolates were identified as *H. pylori* by conventional methods using Gram stain, urease, catalase and oxidase tests, API Campy (Bio Merieux. Cedex. France) and *polymerase chain reaction* (PCR).

#### 2.1.4. Animals

Sixty healthy male Wistar rats (200 ± 3.81 g) obtained from the Pasteur Institute of Algiers (Algeria) were used to evaluate the combined effect of plant extract and probiotic bacteria against *H. pylori*. The animals were kept in individual cages under appropriate environmental conditions (temperature 22 ± 1 °C, 12/12 h light-dark cycle and relative humidity of 60 ± 10%) for two weeks adaptation period at the Veterinary Sciences Institute, Tiaret University. They were housed according to relevant Algerian national legislation, were fed a commercial diet and were given water ad libitum, except otherwise stated. Throughout the experiments, all animals received special veterinary care according to the criteria outlined in the internationally accepted principal guidelines of the European Union on Animal Care (CEE Council 86/609, Directive 63/2010 on the protection of animals used for scientific purposes). The experimental study took place in the animal house of the Veterinary Institute-Tiaret and the experimental protocol was designed and followed by veterinarians. The authors are members of the Algerian Association of Sciences in Animal Experimentation (AASEA) (Agreement Number:45/DGLPAG/DVA.SDA.14).

### 2.2. Methods

#### 2.2.1. Polymerase Chain Reaction Identification of *H. pylori*

Genomic DNAs were extracted from all strains by the guanidinium thiocyanate method. For the PCR detection of *H. pylori*, specific primers for the *ureC* gene have been used [35].

#### 2.2.2. Evaluation of Anti-*H. pylori* Effect of Plant Extracts

##### Evaluation of the Diameter of Inhibition Zones (DIZ) of Plant Extracts Using Disc Diffusion Method

The disc diffusion test was used for primary screening of the susceptibility of *H. pylori* to the plant extracts. Bacterial suspensions adjusted to McFarland turbidity standard 0.5 (1.5 × 10^−8^ CFU/mL) were inoculated on plates containing Muller-Hinton agar with 5% of horse blood. Filter paper discs (6 mm diameter) impregnated with 60 μL of different concentrations of each plant extract (10, 20, 30, 40, 50, 60, 70, 80, 90, 100, 150, 250, 500 and 1000 μg/mL) were placed on the inoculated agar surfaces. Methanol at a concentration of 80% (60 μL) was used as a negative control. The plates were observed for 2 to 3 days at 37 °C under microaerophilic conditions. The antibacterial activity was expressed as the mean of inhibition diameters (mm) produced by the plant extracts [36].

##### Evaluation of the Minimum Inhibitory Concentration (MIC) of Plant Extracts with the Agar Dilution Method

The extracts presenting an inhibition zone ≥9 mm in diameter were chosen to assay the Minimum Inhibitory Concentration (MIC) with the agar dilution method using Mueller Hinton agar with 5% of horse blood. Methanol (80%) was used in the assay as a negative control. Concentrations of each extract were prepared in methanol 80% and 1 mL of each solution was incorporated in 20 mL of appropriate melted agar and poured into a Petri dish. The final concentrations of the extracts in the medium ranged from 90 to 1000 µg/mL. Agar plates were inoculated with 1 mL of bacteria suspension (1.5 × 10^8^ CFU/mL). The plates were incubated for 5–7 days at 37 °C under microaerophilic conditions. The MIC was defined as the lowest concentration of plant extract inhibiting visible growth [37].

##### Evaluation of the Minimum Inhibitory Concentration (MIC) and Minimum Bactericidal Concentration (MBC) of Plant Extracts with the Broth Dilution Method

Minimum Inhibitory Concentrations (MIC) and Minimum Bactericidal Concentration (MBC) were measured using tube dilution methods. Fresh bacterial suspensions were prepared in Mueller Hinton broth and adjusted to McFarland turbidity standard 0.5. Serial concentrations were prepared from crude plant extracts within the range of 90 to 1000 μg/mL; then, 1 mL of each extract was added to tubes containing 8 mL of Mueller Hinton broth. Finally, 1 mL of a 1:1000 dilution of bacteria adjusted was added to obtain a 10 mL final volume. Controls of bacteria without extract were also prepared under similar conditions. The tubes were incubated for 2–3 days at 37 °C before recording the MIC. A volume of 0.1 mL of each suspension was spread onto Columbia agar plates containing 5% horse blood. After incubation in microaerophilic conditions at 37 °C for 72 h, the colonies formed were subsequently computed. The MBC was defined as the lowest concentrations of the plant extract, inducing complete inhibition of colony formation of the test bacteria at the latter cultivation [38].

##### Evaluation of Growth Kinetics of *H. pylori* in the Presence of Plant Extracts

The ability of several plant extracts to suppress *H. pylori* growth was tested by adding 1 mL of each plant extract on the 6th hour of development, while the optical density was subsequently measured every 2 h with UV-Vis spectrophotometer at 600 nm to follow the development of *H. pylori* in the presence of the extract [39].

### 2.3. Evaluation of Anti-H. pylori Effect of Probiotics (Well Diffusion Assay)

#### 2.3.1. Preparation of Cell-Free Supernatant (CFS) of Probiotics

Cell-free culture supernatants of probiotics were prepared as described by Kim et al. [40]. The culture supernatant was collected from a 24 hr culture by centrifugation at 4.000× *g* rpm/10 min. The resulting supernatants were filter-sterilized (pore size 0.22 mm). *H. pylori* cultures were plated on fresh Mueller Hinton agar plates containing 5% horse blood (1.5 × 10^8^ CFU/mL) and wells were drilled into the agar by using sterile Pasteur pipettes; 60 µL of fresh probiotic strains cell-free culture supernatants were introduced in the agar wells. Plates were incubated for 48 to 72 h under microaerophilic conditions at 37 °C and the diameters of inhibition zones around the wells were measured. The antibacterial activity was expressed as the mean of inhibition diameters (mm) produced by each probiotic.

#### 2.3.2. Evaluation of Growth Kinetics of *H. pylori* in the Presence of Probiotics

A pre-culture tube of the *H. pylori* strain was inoculated in Mueller Hinton broth and incubated at 37 °C for 18 h, from which three standardized (0.5 MacFarland) culture tubes were prepared and the measurement of the bacterial growth of the pathogen strain was carried out by measuring the optical density every 2 h with UV-Vis spectrophotometer at 600 nm. After 6 h, 1 mL of each supernatant (normal/organic acid-free/H_2_O_2_ free prepared as described below) was added to the culture tubes; the optical density was then measured every 2 hr up to 24 hr and the OD = f (t) curve was drawn [39].

##### Influence of Organic Acids (Lactic and Acetic Acids)

Lactic acid bacteria can produce inhibitor molecules of *H. pylori,* such as organic acids, bacteriocins and hydrogen peroxide. To ensure the presence of these molecules, we took an 18 h culture of different strains of probiotics which were transferred to 50 mL of modified MRS broth and incubated at 37 °C for 18 h. After incubation, the tubes were centrifuged at 4.000× *g* rpm/10 min in order to recover the supernatant [39].

##### Influence of Bacteriocins

In order to study the effect of bacteriocins on the growth of *H. pylori*, the effect of organic acids, in particular lactic and acetic acids, was eliminated and the supernatant was neutralized (pH = 7) by adding a 0.1 N NaOH solution [39].

##### Influence of Hydrogen Peroxide

The effect of H_2_O_2_ was inhibited by incubating the supernatant fluid with catalase enzyme solutions prepared to a final concentration of 1 mg mL^−1^ in phosphate-buffered saline (pH 7.0).

The inhibitory effect of the agent was tested by eliminating the possible effect of organic acids by adjustment of the cells-free supernatants’ pH to 7 and that of hydrogen peroxide in the presence of catalase.

### 2.4. Combined Effect of Medicinal Plants with Probiotics on H. pylori

#### 2.4.1. Determination of DIZ Using Disc Diffusion Method

Fresh Mueller Hinton agar plates containing 5% horse blood were inoculated with 1.5 × 10^8^ CFU/mL of *H. pylori*; filter paper discs (6 mm diameter) impregnated with 30 μL of fresh supernatants of probiotics +30 μL of plant extracts were placed on the inoculated agar surfaces (each probiotic has been combined with all four extracts). Plates were incubated for 48 to 72 h under microaerophilic conditions at 37 °C and the diameters of inhibition zones around discs were measured. 

#### 2.4.2. Evaluation of Growth Kinetics of *H. pylori* in the Presence of Combined Solutions (Plant Extracts with Probiotics)

Different mixtures of probiotics with plant extracts were examined for inhibition of *H. pylori* growth. The measurement of *H. pylori* growth was carried out by measuring the optical density with UV-Vis spectrophotometer at 600 nm every 2 h after adding 500 µL of probiotic supernatant +500 µL of plant extract on the 6th hour of growth (each probiotic has been combined with all four extracts).

### 2.5. In Vivo Study Protocol

The inhibition of *H. pylori* growth by probiotics and plant extracts was investigated using the Wistar rat model. Depending on the results, we have chosen the strongest probiotic and extract (*B. breve* and fenugreek extract) based on their anti-*H. pylori* effect in order to complete the in vivo study. The rats were divided into 9 groups; 6 groups (G1–G6) were infected for 2 weeks, then treated for the 2 following weeks and 3 preventive groups (G7–G9) were infected and treated orally at the same time once daily for 2 weeks. More precisely, the groups were: Group 1 G1 (NC. n = 6); negative control *H. pylori*-infected 1.5 × 10^8^ CFU without treatment; G2 (PC. n = 6); positive control *H. pylori*-infected and treated using second-line therapy clarithromycin with metronidazole in combination with amoxicillin and omeprazole; G3 (TFE1 n = 6): *H. pylori*-infected and treated with fenugreek extract- TFE) 150 µg/kg; G4 (TFE2. n = 6): *H. pylori*-infected and treated with fenugreek extract 300 µg/kg; G5 (TBB. n = 6): *H. pylori*-infected and treated with *B. breve* (TBB) 1.2 × 10^9^ CFU; G6 (TFE1 + TBB, n = 6): *H. pylori*-infected and treated with fenugreek extract 150 µg/kg and *B. breve* 1.2 × 10^9^ CFU; G7 (HP + TFE, n = 6): *H. pylori* infected and treated with fenugreek extract 150 µg/kg; G8 (HP + TBB. n = 6): *H. pylori* infected and treated with *B. breve* 1.2 × 10^9^ CFU and G9 (HP + TFE + TBB. n = 6): *H. pylori* infected and treated with fenugreek extract 150 µg/kg and *B. breve* 1.2 × 10^9^ CFU. Doses were calculated using the following formula: Injected volume (mL) = [animal weight (kg) x drug dose to be administrated (mg/kg)]/drug concentration (mg/mL).

At the end of the experiment, the rats were euthanized with diethyl ether and a full necropsy was performed.

### 2.6. Histopathologic Analysis of Gastric Tissue Samples

After removal, the stomach of each animal was opened through the longer curvature with sterile surgical instruments. Urease activity was determined in one half and the other half was fixed in 10% buffered formalin, trimmed to include all areas of the stomach, then processed by standard methods [41] and embedded in paraffin. From each block, two 5 µm sections were made, one being stained by hematoxylin and eosin (H&E) and the other by a Giemsa stain for *H. pylori* detection. All cases were examined in a blind manner by a veterinary pathologist, according to criteria established by Lee et al. [42]. Histopathological examination completed for stomach specimens were ranked according to the intensity of *H. pylori* colonization as follows: severe infection (3), moderate infection (2), mild infection (1) and free from infection (0) [43]. Gastritis was defined by the presence of lymphocytic or neutrophilic infiltration.

In Giemsa-stained sections, examining the different anatomical regions of the *H. pylori*-infected and treated rats, *H. pylori* bacteria colonizing the glands were graded on a scale of 0 to 4 as follows: 0, absence of bacteria; 1, bacteria isolated and randomly distributed; 2, reduced number of bacteria; 3, large number of bacteria and 4, very high number of bacteria.

### 2.7. Statistical Analysis

The data were collected and statistically analyzed by use of MS Excel 2007 and presented as mean  ±  SD of three replicates.

## 3. Results and Discussion

### 3.1. Results of Phytochemical Analysis and Screening

Phytochemical analysis showed that phenols are the largest group of metabolites in the four studied plants. Results of phytochemical screening of our plants using HPLC revealed the presence of caffeic acid, isoquercetin, vanillic acid, myricetin 3-0-rutinoside, syringaresinol, citrusine, rosmarinic acid and p-coumaric acid in cumin seeds, gallic acid, sinapic acid, caffeic acid, asterogenic acid, pyrogallol, hyperoside and ferulic acid in fenugreek seeds, gallic acid, quercitin, rutin, hyperoside and karempferol in red onion and one molecule for garlic extract which is gallic acid [32,33]. 

### 3.2. Results of Isolation and Identification of H. pylori

#### 3.2.1. Results of Biochemical Identification

After incubation for 5 days at 37 °C in a microaerophilic atmosphere, the results of the reading were reflected by the appearance of small colonies 1 to 2 mm in diameter. The colonies were grayish or transparent in color, shiny, round and had a regular outline. Microscopic observation in the fresh state showed that *H. pylori* is a small curved, mobile bacillus.

The Gram stain performed on smears from the individual colonies indicated the presence of Gram-negative bacteria. The biochemical tests showed that the strain was positive for urease, catalase and oxidase and therefore had a significant enzymatic activity.

#### 3.2.2. Results of *H. pylori* Identification by PCR 

**Figure 1:**
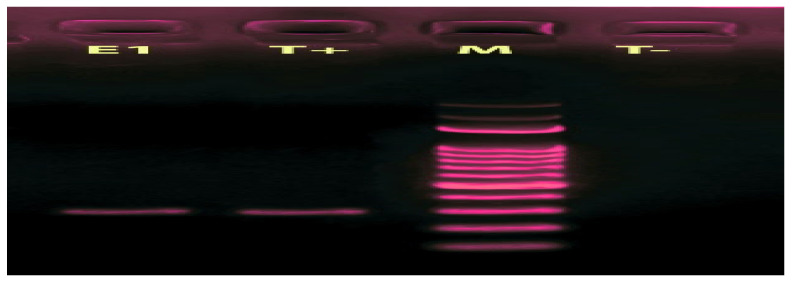
PCR-amplified products of the Ure C gene of *H. pylori* visualized by Gel Red TM at 1.5% of agarose gels analyzed by electrophoresis. E1: sample 1. T+: Positive control (294 bp); T−: Negative control (water); M: size marker (100–3000 bp) (Promega. Madison, WI, USA). 99% *H. pylori* HUP-B14. complete genome (HP).

Real-time PCR assay for the identification of the *H. pylori* isolated strain showed amplification lines for the targeted gene (*ureC*). A 294 bp fragment was obtained in all *H. pylori* isolates after amplification of the *ureC* gene (Figure 1).

Sequence ID: CP003486.1 >TA3_P.UR --16…270 of sequence CAAACCATCGCCGGTTTTAGCGTAATCGCTAAAAATGATATGCCCGCTTGCTCGCCTCCAAAATTGGCTTTATTCAATGCATGCATTCGCTCACAAACTTATCCCCAATCGCGCAATGCTTCAATTCTAAATCTTGGGATTTTAAGTATTCTTTAAGGGCTAAATTACTCATGTTTGTAGCGACAATTGCTTGAGAAGAAAGGGCGTTTTTAGATTTTTGA TAAACCCCTAACACCCCTAAAAGCTTCACCCG.

### 3.3. Results of Evaluation of Anti-H. pylori Effect of the Plant Extracts

#### 3.3.1. Results of Evaluation of *DIZ* of Plant Extracts Using the Disc Diffusion Method

The inhibitory effect of the methanolic extracts on the growth of *H. pylori* is shown in Table 2. The results indicated a concentration-dependent increase in the DIZ against *H. pylori* for all plant extracts. The DIZ started from 6 mm for the different plant extracts at 10 µg initial concentration. High DIZ were recorded for the cumin and fenugreek extracts at 90 µg concentrations and above, with slight primacy of the fenugreek extract (16.00 ± 0.00) at 1000 µg.

#### 3.3.2. Evaluation of Minimum Inhibitory Concentration (MIC) and Minimum Bactericidal Concentration (MBC) of Plant Extracts

The results of plant extracts induced MIC and MBC against *H. pylori* are presented in Table 3. The strongest MIC and the MBC against *H. pylori* were obtained with the fenugreek extract at 100 and 150 µg/mL, respectively. The other extracts rendered as follows: cumin extract 150 µg/mL (MIC) and 250 µg/mL (MBC), while for the onion and garlic extracts, the concentrations needed to be much higher to attain the same MIC and MIBs. This suggested that fenugreek extract provided the best antibacterial effect at the lowest concentrations among all extracts evaluated in the present study.

#### 3.3.3. Results of Evaluation of Growth Kinetics of *H. pylori* in the Presence of Plant Extracts

**Figure 2:**
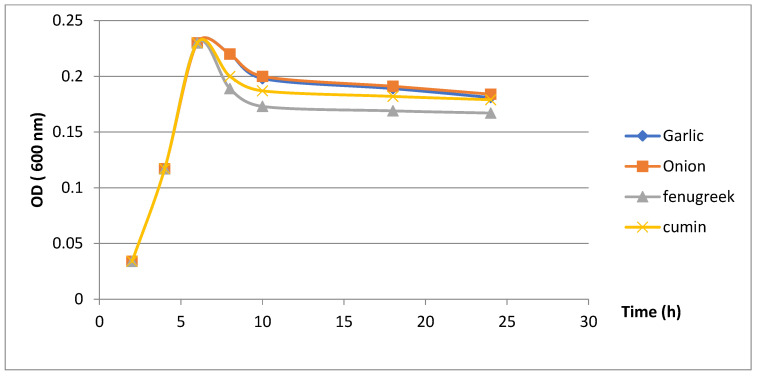
Results of evaluation of growth kinetics of *H. pylori* in the presence of plant extracts.

The inhibition kinetics evaluation showed that plants extract had antibacterial activity against *H. pylori* (Figure 2). Almost similar growth kinetics were recorded for all plant extracts with relatively overlapping curves up to 6 h when adding plant extracts, followed by a marked decrease in H. pylori growth recorded with the fenugreek extract compared to other extracts.

According to the results, fenugreek extract caused the highest growth inhibition of *H. pylori,* followed by cumin, garlic and onion, respectively.

### 3.4. Results of Evaluation of Probiotics Effect on H. pylori

#### 3.4.1. Evaluation of DIZ of Probiotics against *H. pylori* Using Well Diffusion Assay

The antibacterial activity of 12 probiotic strains was evaluated in vitro by the well diffusion method against *H. pylori*. Table 4 summarizes the microbial growth inhibition of *H. pylori* in the presence of probiotics’ supernatants. While most probiotic strains (*Lactobacillus* genus) showed the mean diameter of inhibition zones (DIZ) ranging from 10 to 10.67 mm (*L. rhamnosus LA80*, *L. rhamnosus GG*, *L. helviticus*, *L. lactis* and *S. thermophilus*), a very high DIZ (20.33 ± 0.58 mm) was recorded for *B. breve* strain.

#### 3.4.2. Results of Evaluation of Growth Kinetics of *H. pylori* in the Presence of Probiotics

**Figure 3:**
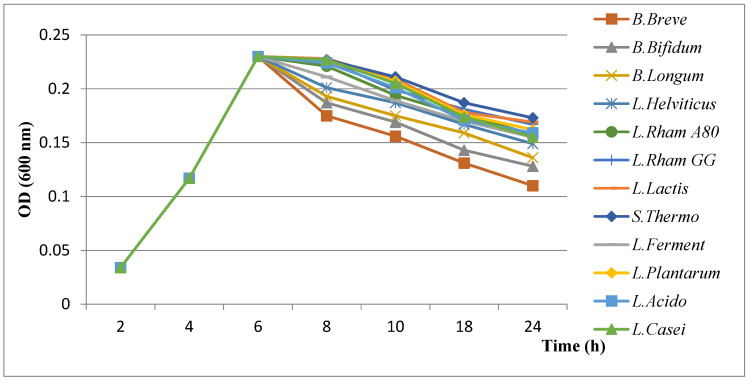
Results of evaluation of growth kinetics of *H. pylori* in the presence of supernatant of probiotics.

**Figure 4:**
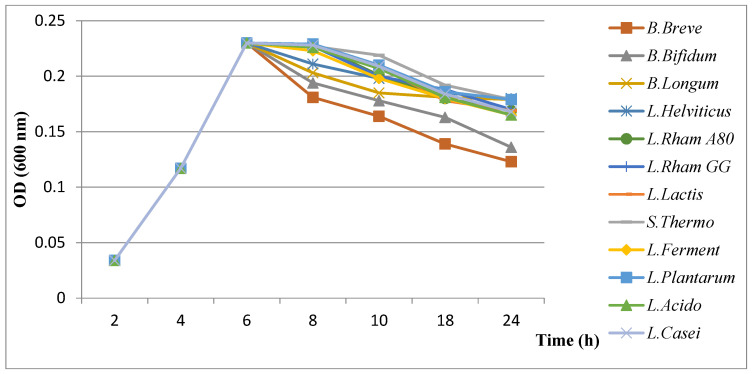
Results of evaluation of growth kinetics of *H. pylori* in the presence of neutralized supernatant of probiotics.

**Figure 5:**
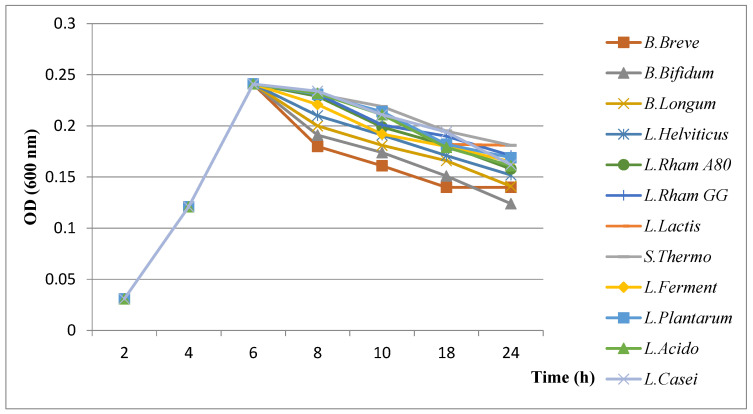
Results of evaluation of growth kinetics of *H. pylori* in the presence of supernatant of probiotics+ catalase.

The results of evaluation of the growth kinetics of *H. pylori* showed a slight decrease in the presence of probiotics (Figure 3, Figure 4 and Figure 5). An important inhibition effect was recorded when the cell-free supernatant obtained from a culture of probiotics was used, indicating that a diffusible molecule (organic acids) should be present in this supernatant. However, a slightly lower inhibitory effect was obtained using bacteriocin-free and H_2_O_2-_free (neutralized) supernatants.

✓ Influence of organic acids (lactic and acetic acids)

Among the tested probiotics, *B. breve* was responsible for an important growth decrease in *H. pylori* that could be explained by the high production of organic acids.

✓ Influence of bacteriocins

Looking at the inhibition by organic acids, a highly reduced growth rate of *H. pylori* was induced by *B. breve*, suggesting its ability to synthesize more bacteriocins than the other tested probiotics.

✓ Influence of hydrogen peroxide (H_2_O_2_)

No significant changes were observed with the suppression of H_2_O_2_ activity for all tested bacteria suggesting that it has no effect on *H. pylori* growth.

### 3.5. Results of Combined Effects of Medicinal Plants with Probiotics on H. pylori

#### 3.5.1. Quantifying the DIZ Using the Disc Diffusion Method

Results of the determination of DIZ of medicinal plants combined with probiotics against *H. pylori* are shown in Table 5). The combined effect of most probiotics with plant extracts is quite similar, except for *B. longum*, *B. bifidum* and *B. breve* showing highly distinct DIZ values. The highest DIZ values were obtained with all *B. breve* combinations, especially with fenugreek extract giving 28.67 ± 0.58 mm DIZ against the *H. pylori* strain.

#### 3.5.2. Evaluation of Growth Kinetics of *H. pylori* in the Presence of Combined Extract Plants with Probiotics

**Figure 6:**
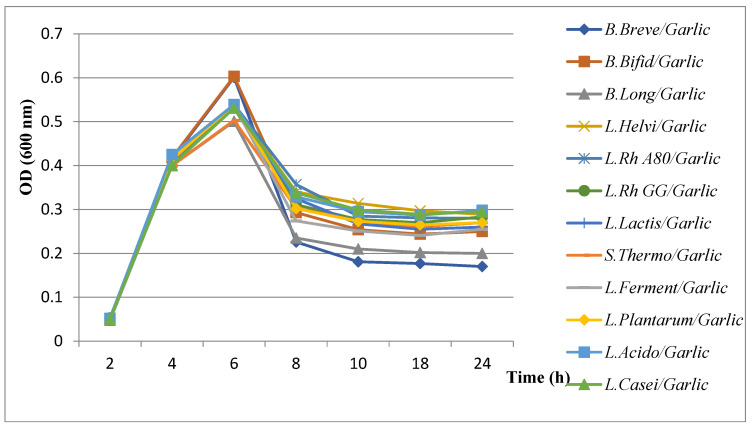
Results of evaluation of growth kinetics of *H. pylori* growth kinetics in the presence of probiotics supernatant + garlic extract.

**Figure 7:**
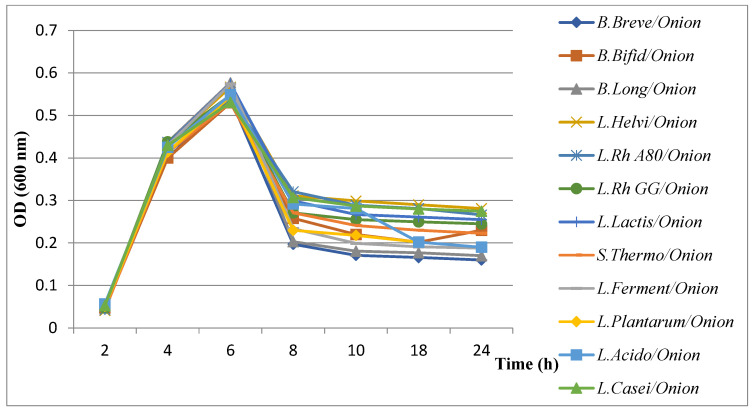
Results of evaluation of growth kinetics of *H. pylori* growth kinetics in the presence of probiotics supernatant + onion extract.

**Figure 8:**
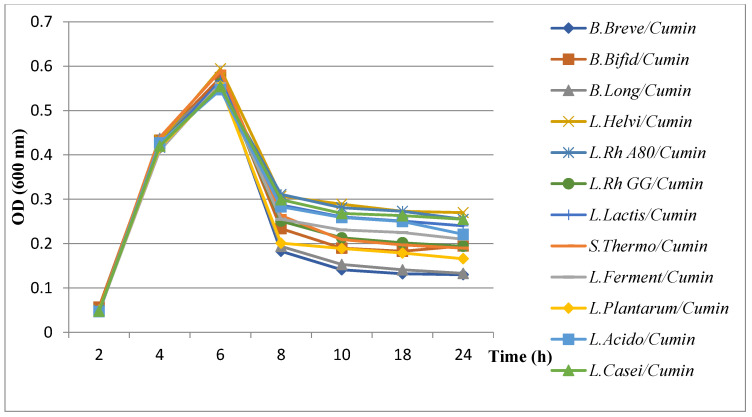
Results of evaluation of growth kinetics of *H. pylori* growth kinetics in the presence of probiotics supernatant + cumin extract.

**Figure 9:**
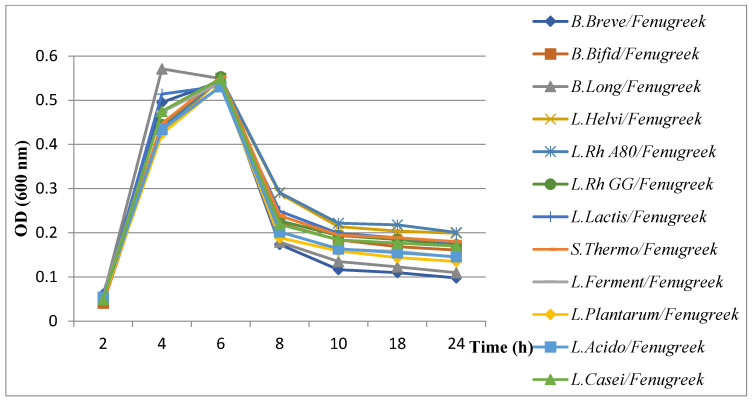
Results of evaluation of growth kinetics of *H. pylori* growth kinetics in the presence of probiotics supernatant + fenugreek extract.

The results of the evaluation of *H. pylori* growth kinetics showed a remarkable decrease in the presence of probiotics combined with plant extracts (Figure 6, Figure 7, Figure 8 and Figure 9). All probiotics have induced an important decrease in the growth of *H. pylori* in combination with plant extracts. However, the most notable decrease in growth was recorded with the fenugreek extract when combined with *B. breve* (Figure 8).

### 3.6. Results of the In Vivo Study

During the acclimation and the experimental periods, most of the rats did not show any treatment-dependent clinical signs, except those in group 9 (HP + TFE + TBB), where severe weight loss that could be attributed to the number of gavage (three consecutive gavages per day) was observed. No gross lesions were observed during the necropsy at the end of the experimental period.

**Figure 10:**
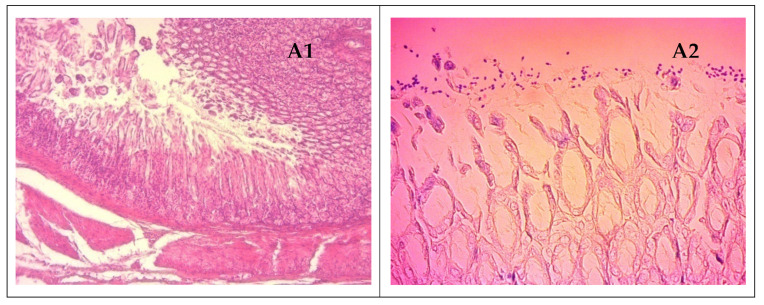

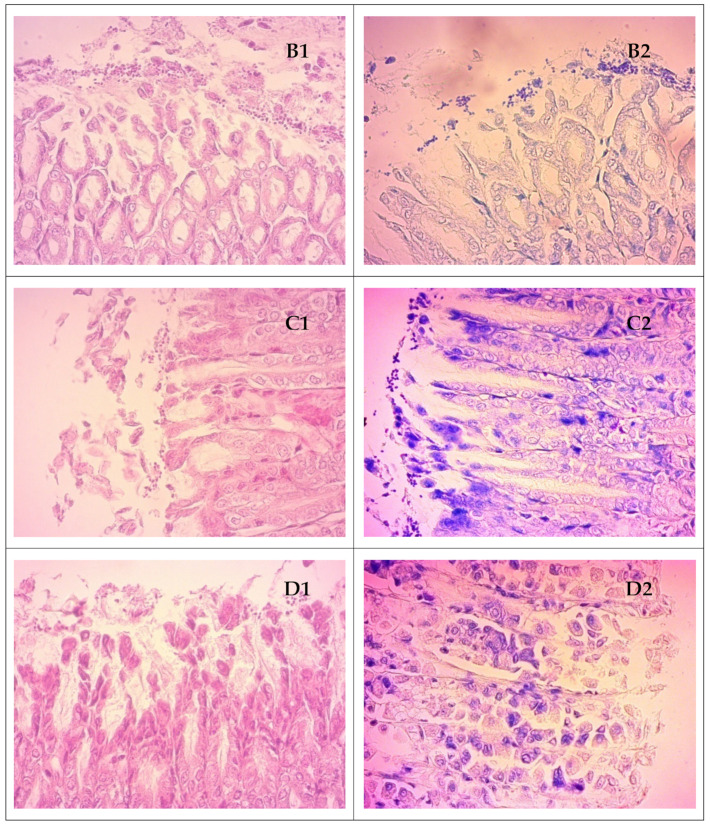
Histopathologic evaluation of antral gastric samples from *H. pylori*-infected rat groups, A non-treated showing moderate inflammatory infiltration in the *lamina propria* ((**A1**): H&E, ×100); note the severe colonization of antral mucosa with *H. pylori* bacteria ((**A2**): Giemsa stain, ×400); B, gastric samples of animals treated with second-line therapy showed reduced number of inflammatory cells in the *lamina propria* ((**B1**): H&E, ×400), no changes of colonizing bacteria were observed ((**B2**): Giemsa stain, ×400); C, treatment with TFE2 and TBB have significantly reduced the severity of inflammatory infiltration ((**C1**): H&E, ×400) with slight reduction in bacterial colonization ((**C2**): Giemsa stain, ×400); D, tissue from animals in all preventive groups showed normal gastric histology ((**D1**): H&E, ×400), with significant reduction in bacterial colonization ((**D2**): Giemsa stain, ×400).

The histological results obtained by administering the combination between *B. breve* and fenugreek extract in order to evaluate their effect on *H. pylori*-induced stomach inflammation in vivo were shown in Figure 10.

### 3.7. Discussion

*H. pylori* isolated from biopsy samples of gastric ulcer patients were identified by different tests as described above. The detection of the *ureC* gene of the *H. pylori* strain was further confirmed by PCR amplification of the *ureC* gene specific to the strain of *H. pylori*. Our results of PCR were similar to the previous study by Lage et al., (1995) that showed the amplification of curves for the targeted gene (*ureC*) [35].

The evaluation of the antibacterial effect of methanolic extracts from tested plant materials against *H. pylori* clinical isolates using a disc diffusion test and by evaluating the MIC on solid media and MBC on broth media indicated an inhibitory activity in high percentages. The results presented in this study emphasize the significant antibacterial effect of methanol extracts, especially for fenugreek extract, which was higher than that of cumin, onion and garlic extracts, respectively; all tested plants showed substantial but broadly different anti-*H. pylori* effects with an MBC ranging from 150 to 1000 µg/mL.

Similar results were obtained by other investigators for anti-*H. pylori* activities of the studied plants. Thus, O’Gara et al. [17] and Cellini [44] showed that garlic exhibited a potential anti-*H. pylori* effect with MIC ranging from 250 to 500 μg/mL and a MBC ranging from 250 to 500 μg/mL [17,44]. The in vitro anti-*H.pylori* activity of extracts and compounds obtained from garlic has been extensively documented [44,45]. An aqueous garlic extract had a MIC of 40 μg/mL against *H. pylori*, and for other garlic compounds (allicin, ajoenes, vinyldithiins, thiosulphinates), the MIC values were approximately 10 to 25 μg/mL [45,46]. Similarly, the other plants studied in this research represented subject to investigations of other groups. Onion extracts presented a good anti-*H. pylori* activity, according to Yordanov et al. [18], while cumin showed a MIC of 691 μg/mL against *H. pylori* [19] and, finally, fenugreek was observed to moderately (68%) inhibit the growth of *H. pylori* according to Manjegowda and Dharmesh [20]. The bactericidal activity of plant preparations against *H. pylori* significantly depends on the type of the extract and its components, concentration and exposure time and the density of the tested bacterial strains [47]. In previous studies, phytochemical screening of four plants was carried out and the results revealed the presence of eight phytochemical compounds in cumin extract, seven molecules in fenugreek extract, five compounds found in red onion extracts and one compound in garlic [32,33]. From these phytochemical studies, phenols are the largest group of metabolites in the studied plants and many compounds are believed to have remarkable anti-*H. pylori effect* in vitro and, to some extent, in vivo. Studies that have attempted to elucidate the mechanism of action of plant extracts or compounds on *H. pylori* are available. Some of these studies, for example, investigated the effect of various compounds on bacterial virulence factors such as urease, adhesion, vacuolization, motility or on specific metabolic enzymes [48]. The antimicrobial activity of the secondary metabolites of plants is related to the -OH group bound to the phenolic ring [49].

The anti-*H. pylori* mechanism of action of some of the phytochemicals listed above is described. Some of the phenolic acids demonstrated antimicrobial activity similar to antibiotics [50]. For instance, quercetin showed activity against several bacteria causing inhibition of FabZ enzyme [51] and, in the same way, to kaempferol by inhibition of peptidoglycan synthesis, activity of β-lactamase, decrease in fatty acids and increase in cytoplasmic membrane permeability [52]. Caffeic acid is able to rupture cell membranes, interfere with aerobic metabolism [53] and inhibit efflux pump [54]. In a separate study, several phenolic acids, including vanillic acid, syringic acid, p-coumaric acid and ferulic acid, from Polish wild mushrooms demonstrated intermediate antibacterial activities against a range of Gram-negative and Gram-positive bacteria [55]. Hyperoside, ferulic acid, rutin and ellagic acid found in the extracts of three species of Potentilla displayed a good anti-bacterial activity against both Gram-positive and Gram-negative bacteria [56].

The anti-*H. pylori* activity in vitro of garlic extracts and compounds has been extensively reported; for example, the gallic acid potentially enhanced the activity of antibiotics against several *P. aeruginosa* isolates by altering the cell wall integrity [57]. In their study, Borges et al., found that ferulic acid and gallic acid had antimicrobial activity against four pathogenic bacteria by producing local damage and leakage (pore formation) of cellular components [58].

Out of the tested probiotic strains, *Bifidobacterium breve* showed the highest antibacterial activity against *H. pylori*. Moreover, the antibacterial activity of the other *Bifidobacterium strains*, *B. bifidum* and *B. longum,* was also higher than that of the reference strains (*L. rhamnosus GG*, *L. rhamnosus LA80* and *L. helviticus*) and of isolated probiotic strains (*L. fermentum*, *L. plantarum*, *L. casei*, *L. acidophilus*, *L. lactis* and *S. thermophilus*). Further studies on the inhibition kinetics showed that *B. breve* presents the highest antibacterial activity against *H. pylori* when compared to other probiotic strains. Regarding the results of growth kinetics of *H. pylori* in the presence of probiotics, when investigating the influence of different variables (organic acids, bacteriocins and hydrogen peroxide), it was clear that the inhibition effect was due to the presence of organic acids, such as lactic and acetic acids and bacteriocins. Thus, in accordance with the bacterial death criteria of Pearson et al. [59], the effect of *L. fermentum* UCO-979C strain at 24 h can be considered lethal for *H. pylori*. Further, after repeating this assay at shorter times and using the same Pearson criteria for interpretation of the results, the death of H. pylori after 6 h of treatment with B. breve was noticed. Nevertheless, other researchers found different results when evaluating the anti-*H. pylori* effect of some probiotics. Bae et al., investigated the inhibitory effects of different *Bifidobacterium* spp. on the growth of *H. pylori* and indicated that significant suppression of *H. pylori* growth occurred, especially in the presence of *B. breve* [60]. García et al., establish that *L. fermentum*, *L. casei* and *L. rhamnosus* induced an inhibition ranging from 2 to 5 mm [30]. Boyanova et al. [61], when looking at the anti-*H. pylori* effect of some *Lactobacillus* strains, indicated that three strains suppressed the growth of >86% of *H. pylori* strains at low pH values and two strains suppressed the growth of >53% of the test strains at neutral pH values [61]. Further studies by Boyanova et al. [62] revealed that the DIZ of some *Lactobacillus* strains were higher, from 13 to 16 mm, while Chen et al. [63] found that *L. rhamnosus* and *L. acidophilus* exhibited an inhibitory effect against *H. pylori* reference strain 26695 demonstrated by a DIZs of 12.3 and 11.3 mm, respectively [62,63]. However, Paucar-Carrión et al. [61], when analyzing the anti-*H. pylori* activity of L. fermentum, concluded that the activity was mild with DIZ between 1 mm and 2 mm [64].

Among probiotics, *Bifidobacterium* is one of the favorites, is generally used for the prevention of gastrointestinal infection and it is commonly incorporated in fermented dairy products or food supplements. *Bifidobacterium* exerts an in vitro anti-*H. pylori* effect and inhibits adhesion to the mucosa by competitive exclusion [65]. Several studies have demonstrated a direct relationship between the addition of potential probiotic strains and the in vitro inhibition of *H. pylori* growth. Such strains were *Lactobacillus acidophilus* [66] and *Lactobacillus casei* Shirota strain [26], which, among others, have an antagonistic effect on *H. pylori.* Since most organic acids are produced by the *Bifidobacterium* spp. inhibited the growth of *H. pylori*, while the *H. pylori* growth was not inhibited by organic acids produced in some other *Bifidobacteria*-cultured media, it was suggested that those *Bifidobacterium* strains might produce antibiotic-like compounds (bacteriocins) [60]. The results of the present study confirm the findings of Zacharof and Lovitt [67], which imply the effects of organic acids and other bioactive substances of the *Bifidobacterium* and *Lactobacillus* strains and a higher activity of bacteriocins at lower pH values [67]. The activity of the neutralized and catalase-treated supernatants was approximately similar with regard to the *H. pylori* growth inhibition effect. Bacteriocin activity depended on numerous factors of the test strains, such as the bacterial cell envelope/membrane composition, reduction in bacteriocin binding/insertion, bacteriocin sequestering and degradation or efflux pumping [68].

In this study, we confirmed in in vitro experiments the inhibitory activity of *B*. *breve* complex mixture containing fenugreek extracts on *H. pylori*. The fenugreek extract inhibited the growth of *H. pylori* in a dose-dependent manner (100 µg/mL). In addition, the inhibitory effect on *H. pylori* of *B. breve* and fenugreek extract, when applied as a complex mixture rather than individual components, was confirmed to be superior. 

*H. pylori* was inhibited by all combined mixtures of extracts and probiotics with varying results, while fenugreek/*B. breve*, cumin/*B. breve*, garlic/*B. breve* and onion/*B. breve* combinations exhibited relevant anti-*H. pylori* activities with DIZ of 26, 29, 23 and 25 mm, respectively. Preliminary studies on the effect of probiotics against *H. pylori* revealed that inhibition may be due to lactic acids and bacteriocins. However, the presence of phenolic compounds, such as gallic acid, caffeic acid, quercetin and vanillic acid, in plant extracts may also have an influence.

Few studies investigate the antimicrobial effects of combinations between probiotics and plant extracts. Lee et al. [69] demonstrated that the treatment with a complex mixture of *L. paracasei* HP7, including the extract of *P. frutescens* and *G. glabra,* could inhibit the growth of *H. pylori* and, thus, is a promising treatment for patients with gastric symptoms, such as gastritis caused by *H. pylori* infection [69]. Behrad et al. [70] recorded that a mixture of cinnamon extract, *L. acidophilus LA-5* and NCFM, *Bifidobacterium* Bb-12, *L.casei* LC-10 and *Streptococcus thermophilus* Th-4 exhibited the strongest inhibitory effect on *H. pylori* growth in vitro (13.5 mm) [70]. Other studies contradict the synergic effect between plant extracts and probiotics. Kang et al., (2021) investigated the anti-*H. pylori* effects of *Lactobacillus plantarum* (pH3A), monolaurin, grapefruit seed extract and their synergies in vitro and in vivo [71]. Monolaurin and grapefruit seed extract suppressed *H. pylori* growth at a MIC of 62.5 ppm. Additionally, *L. plantarum* pH3A significantly inhibited *H. pylori* growth. In the in vivo study, *H. pylori* colonization of the mouse stomach was significantly reduced by *L. plantarum* treatment, but the addition of monolaurin or grapefruit seed extract did not contribute to these anti-*H. pylori* activities. Therefore, the *L. plantarum* strain can potentially be applied as an alternative anti-*H. pylori* therapy, but evidence of its synergy with monolaurin or grapefruit seed extract in vivo is still lacking. Sadeghi et al. [72] examined the synergistic effect of broccoli sprout extract with probiotics on *H. pylori* growth inhibition and the findings showed a synergistic effect of the mixture on bacterial inhibition and suggested the use of broccoli sprout extract and probiotic bacteria in a yogurt form that is effective in the therapy of *H. pylori* infection [72].

The histopathological examination of gastric mucosa showed a moderate inflammatory infiltration in the *antrum* and *fundus* of animals in the non-treated groups infected with *H. pylori* and severe colonization of antral mucosa with *H. pylori* bacteria by use of Giemsa stain. Gastric samples of *H. pylori*-infected animals treated with second-line therapy showed a reduced number of inflammatory cells in the *lamina propria*; however, no changes in colonizing bacteria were observed in the Giemsa-stained samples. Tissue samples of rats infected with *H. pylori* and treated with TFE2 and TBB showed a significant decrease in infiltrating inflammatory cells and colonizing bacteria scores (1–0 and 1–1) for inflammation and colonization, respectively. However, the group of rats receiving the lowest dose of fenugreek extract (TFE1, 150 mg/kg) showed a slight decrease in inflammatory infiltration and colonization of gastric mucosa (score 2–2) compared to the higher dose group animals.

All animals in preventive groups showed normal gastric histology with a significant reduction in bacterial colonization (score 1). However, the gastric mucosa of treated animals exhibited mild inflammation due to infiltration by lymphocytes from previous *H. pylori* infections. Animals in the *H. pylori*-infected group treated with fenugreek extract associated with *B. breve* showed normal gastric histology with a total absence of *H. pylori* characteristic forms of colonialization in Giemsa-stained samples.

Data from the current study suggest that administration of TFE, TBB or their associations to *H. pylori*-infected rats can moderately reduce the number of *H. pylori* colonizing the gastric mucosa with an important effect on associated gastritis. However, prophylactic administration of TFE, TBB or their associations could prevent gastric inflammation and considerably reduce or completely eliminate *H. pylori* colonization.

In vivo anti-*H. pylori* activity of the fenugreek extract and/or *B. breve* has been confirmed by histopathological and urease tests. These compounds have suggestively reduced the number of animals with a positive urease test and the number of detected microorganisms in histological sections stained by Giemsa, exceedingly even the standard medications, thus preventing the presence of these organisms in the gastric mucosa.

None of the animals in the control and treated groups exhibited atrophy or metaplasia of the gastric mucosa. Similarly, important differences in the antral and fundic mucosa with respect to colonization by *H. pylori* and the associated gastritis were observed between the treated and control groups, as also reported by Sgouras et al. [26]. It has been suggested in previous in vivo studies in rats that the fenugreek extract forms a protective barrier over the gastric epithelial lining due to the presence of galactomannans, *H. pylori* possibly interacting with epithelial cells through secretory molecules (vitexin-7-O-glucoside, vicenin-1, luteolin and orientin) or as a result of adherence [73].

The significant reduction in the severity of gastritis observed in animals treated with the extract indicated a possible role as an anti-inflammatory and anti-secretory agent [73,74]. However, this gastro-protective action was not attributed exclusively to the alteration of gastric pH or acidity since the response of the extract was consistent in both in vivo and in vitro studies [73]. Additionally, it has been suggested that the in vivo activity of the extract may not result solely from the topical action but may also take place from a systemic component [75]. 

Several probiotics showed beneficial effects in animal models of *H. pylori* infection. In mice infection models, a combination of probiotics—*L. acidophilus* R0052 + *L. rhamnosus* R0011 and *L. casei* Shirota strain + *L. johnsonii* strain La1—were found to reduce the effects of *H. pylori* infection by decreasing *H. pylori* colonization and alleviating *H. pylori*-induced inflammation of the gastric mucosa [26,27].

There are numerous possible mechanisms by which probiotic bacteria can prevent *H. pylori* adhesion [76]. *Lactobacilli,* such as *L. johnsonii* La1 [77] or *L. acidophilus* LB [78], may exert anti-adhesion activity by secreting antimicrobial substances and strains, such as *L. reuteri,* can inhibit *H. pylori* growth by competing with adhesion sites. Non-specific rather than specific receptor site blockage is the most likely mechanism [79].

In vivo studies demonstrated that previous colonization with probiotics prevents or reduces *H. pylori* infection in experimental animals [27]. Consequently, regardless of the mechanisms of inhibition of the *H. pylori* adherence to epithelial cells, probiotics could prevent *H. pylori* colonization by inhibiting bacterial adhesion to gastric epithelial cells [80].

## 4. Conclusions

The administration of a complex mixture of *Bifidobacterium breve* and *T. foenum-graecum* L. extract was more effective than that of *B. breve* alone or *T. foenum-graecum* L. extract alone, the combination induced a higher antibacterial effect against *H. pylori* and prevented the inflammation induced by *H. pylori*. The present study confirmed that the administration of this mixture reduced the damage of the gastric mucous membrane. The mechanism probably relied on the inhibitory effect of *B. breve* on the adhesion of *H. pylori* to the gastric mucosa combined with the increased secretion of gastric mucosal mucin induced by *T. foenum-graecum* L. extract. Thus, a complex mixture of *B. breve*, including the extract of *T. foenum-graecum* L., could be used to treat patients with gastric symptoms, including ulcers caused by *H. pylori*. These results demonstrated that the complex mixture of *B. breve*, including the extract of *T. foenum-graecum* L. is a promising therapeutic agent for patients with gastric symptoms, such as for gastroduodenal diseases caused by *H. pylori* infection.

## Figures and Tables

**Table 1:** Source and origin of probiotic strains used in the present study.

Probiotic Strains	Source	Origin
*Lactobacillus acidophilus*	Local dairy product	Laboratory of Sciences and Technics of Animal Production, Mostaganem, Algeria
*Limosilactobacillus* *fermentum*	Local dairy product	
*Lactiplantibacillus* *plantarum*	Local dairy product	
*Lacticaseibacillus* *casei*	Local dairy product	
*Lactococcus lactis*	Local dairy product	
*Streptococcus thermophilus*	Local dairy product	
*Lacticaseibacillus* *rhamnosus LA180*	LACTIBIANE	Pharmacy, Lille, France
*Lacticaseibacillus* *rhamnosus GG*	PROBIOLOG	
*Lactobacillus helviticus*	LAXATRANSIT	
*Bifidobacterium longum*	BENEFLORA	
*Bifidobacterium bifidum*	BENEFLORA	
*Bifidobacterium breve*	Local dairy product	Laboratory of Natural and Local Bioresources, University of Hassiba Benbouali, Chlef-Algeria

**Table 2:** Results of determination of DIZ of plant extracts against *H. pylori*.

	DIZ (mm)	Garlic Extract	Onion Extract	Cumin Extract	Fenugreek Extract
Concentrations (µg)					
10		6.00 ± 0.00	6.00 ± 0.00	6.00 ± 0.00	6.00 ± 0.00
20		6.00 ± 0.00	6.00 ± 0.00	6.00 ± 0.00	6.00 ± 0.00
30		6.00 ± 0.00	6.00 ± 0.00	6.00 ± 0.00	6.00 ± 0.00
40		6.67 ± 0.58	6.67 ± 0.58	6.67 ± 0.58	6.67 ± 0.58
50		7.00 ± 0.00	7.00 ± 0.00	7.00 ± 0.00	7.00 ± 0.00
60		7.33 ± 0.58	7.33 ± 0.58	7.33 ± 0.58	7.33 ± 0.58
70		7.67 ± 0.58	8.00 ± 0.00	8.00 ± 0.00	8.33 ± 0.58
80		8.00 ± 0.00	8.33 ± 0.58	8.67 ± 0.58	9.00 ± 0.00
90		8.33 ± 0.58	8.67 ± 0.58	9.33 ± 0.58	9.67 ± 0.58
100		9.00 ± 0.00	7.79 ± 0.58	10.33 ± 0.58	10.67 ± 0.58
150		9.67 ± 0.58	10.67 ± 0.58	11.00 ± 1.00	11.33 ± 1.15
250		10.33 ± 0.58	11.33 ± 0.58	12.33 ± 0.58	12.67 ± 0.58
500		11.67 ± 0.58	12.67 ± 0.58	14.33 ± 0.58	14.67 ± 0.58
1000		13.33 ± 0.58	14.67 ± 0.58	15.67 ± 0.58	16.00 ± 0.00

**Table 3:** Results of determination of MIC and MBC of plant extracts against *H. pylori*.

Plant Extract	Garlic	Onion	Cumin	Fenugreek
MIC (µg/mL)	500	250	150	100
MBC (µg/mL)	1000	500	250	150

**Table 4:** Results of evaluation of *DIZ* provided by probiotics against *H. pylori*.

Probiotic Strains	DIZ (mm)
*B. breve*	20.33 ± 0.58
*B. bifidum*	12.67 ± 0.58
*B. longum*	11.33 ± 0.58
*L. rhamnosus LA80*	10.67 ± 0.58
*L. rhamnosus GG*	10.67 ± 0.58
*L. helviticus*	10.00 ± 0.00
*L. lactis*	10.67 ± 0.58
*S. thermophilus*	10.33 ± 0.58
*L. plantarum*	10.67 ± 0.58
*L. acidophilus*	10.67 ± 0.58
*L. fermentum*	10.33 ± 0.58
*L. casei*	10.00 ± 0.00

**Table 5:** Results of determination of DIZ of medicinal plants with probiotics against *H. pylori*.

	DIZ (mm)	Garlic	Onion	Cumin	Fenugreek
Probiotic Strains					
*B. breve*		22.67 ± 0.58	24.67 ± 0.58	26.67 ± 0.58	28.67 ± 0.58
*B. bifidum*		14.33 ± 0.58	15.67 ± 0.58	16.33 ± 0.58	17.67 ± 0.58
*B. longum*		13.67 ± 0.58	14.33 ± 0.58	15.33 ± 0.58	16.33 ± 0.58
*L. rhamnosusLA80*		11.67 ± 0.58	12.67 ± 0.58	13.67 ± 0.58	14.33 ± 0.58
*L. rhamnosusLA80GG*		11.67 ± 0.58	12.00 ± 0.00	12.33 ± 0.58	13.00 ± 0.00
*L. helveticus*		11.00 ± 0.00	11.67 ± 0.58	12.00 ± 0.00	12.33 ± 0.58
*L. lactis*		12.00 ± 0.00	12.33 ± 0.58	12.67 ± 0.58	13.67 ± 0.58
*S. thermophilus*		11.67 ± 0.58	12.33 ± 0.58	13.33 ± 0.58	13.67 ± 0.58
*L. plantarum*		11.33 ± 0.58	12.33 ± 0.58	13.00 ± 0.00	13.67 ± 0.58
*L. acidophilus*		11.00 ± 0.00	11.67 ± 0.58	12.00 ± 0.00	12.67 ± 0.58
*L. fermentum*		11.33 ± 0.58	12.33 ± 0.58	13.00 ± 0.00	13.33 ± 0.58
*L. casei*		11.33 ± 0.58	12.33 ± 0.58	13.33 ± 0.58	13.67 ± 0.58

## Data Availability

Not applicable.

## Supplementary Materials

The following supporting information can be downloaded at: https://www.mdpi.com/article/10.3390/microorganisms11051242/s1, The molecular separation of garlic, red onion, fenugreek and cumin methanolic extracts was achieved by HPLC at three wavelengths: 254 nm, 326 nm and 360 nm. The findings obtained are visible in the peaks and retention time of chromatograms of each molecule. The results obtained are shown in the chromatograms with peaks and retention time of each molecule (Figures S1–S4) [32,33]. HPLC results revealed the presence of five components in red onion extract (Figure S1), one component in garlic extract (Figure S2), fifteen compounds in cumin extract (Figure S3) and eight compounds in fenugreek (Figure S4), The identification of molecules found in the samples is based on comparing their retention times (Rt) with that of pure standards under the same experimental conditions (Table S1).
