# Supplementary material for: In Vitro and In Vivo Study of Combined Effect of Some Algerian Medicinal Plants and Probiotics against Helicobacter pylori
Source: Microorganisms. 2023 May 8;11(5):1242. doi: 10.3390/microorganisms11051242 (PMC10224446; doi:10.3390/microorganisms11051242)
Supplement: Supplementary file 1 [file microorganisms-11-01242-s001.zip › microorganisms-2339423-supplementary.pdf]

Chromatographic analysis (HPLC)

The molecular separation of garlic, red onion, fenugreek and cumin methanolic extracts was achieved by HPLC at three wavelengths: 254nm, 326nm and 360nm. The findings obtained are visible in the peaks and retention time of chromatograms of each molecule. The results obtained are shown in the chromatograms with peaks and retention time of each molecule (Figures S1–S4).

HPLC results revealed the presence of five components in red onion extract (Figure S1), one component in garlic extract (Figure S2), fifteen compounds in cumin extract (Figure S3) and eight compounds in fenugreek (Figure S4),The identification of molecules found in the samples is based on comparing their retention times (Rt) with that of pure standards under the same experimental conditions (Table S1).

Chromatographic analysis of the samples identified five phytochemical molecules for red onion extract namely: gallic acid, quercitin, rutin, hyperoside and karempferol and one molecule for garlic extract which is gallic acid. On the other hand eight phytochemical compounds could be identified in cumin extract, namely: caffeic acid, isoquercetin, vanillic acid, myricetin 3-0, rutinoside, syringaresinol, citrusine, rosmarinic acid, p-coumaric acid. Seven compounds of fenugreek extract are: gallic acid, sinapic acid, caffeic acid, asterogenic acid, pyrogallol, hyperoside and ferulic acid. The rest of the compounds that appeared on the chromatograms could not be identified.

The polyphenols separated from the red onion extract at retention times of 3.137 min and 3.687 min are of the tannin class, probably Gallic acid derivatives, according to the spectra and absorption maxima.

Flavonoids are isolated from the same extract at retention times of over 10 min, with the ones from 14.734; 16.451; and 17.967 min being probably Quercetol derivatives with maximum absorption at over 350 nm. Among the majority flavonoids in the red onion extract, the flavonoid from the minute 14.734 represents 48.7%. The flavonoid from minute 10.728 represents 26.5 %, with the rest being in the proportion of less than 10%. There are not many polyphenols in the garlic extract. The only observable component of minute 5.904 is in very low concentration.

Under the same experimental conditions, a comparison of the retention times (Rt) of molecules found in the samples with those of pure standards identified five compounds in the methanolic extracts of red onion (Gallic acid, Quercitin, Rutin, Hyperoside, and Karempferol), as well as one compound in garlic (Gallic acid) and two other compounds that could not be identified.

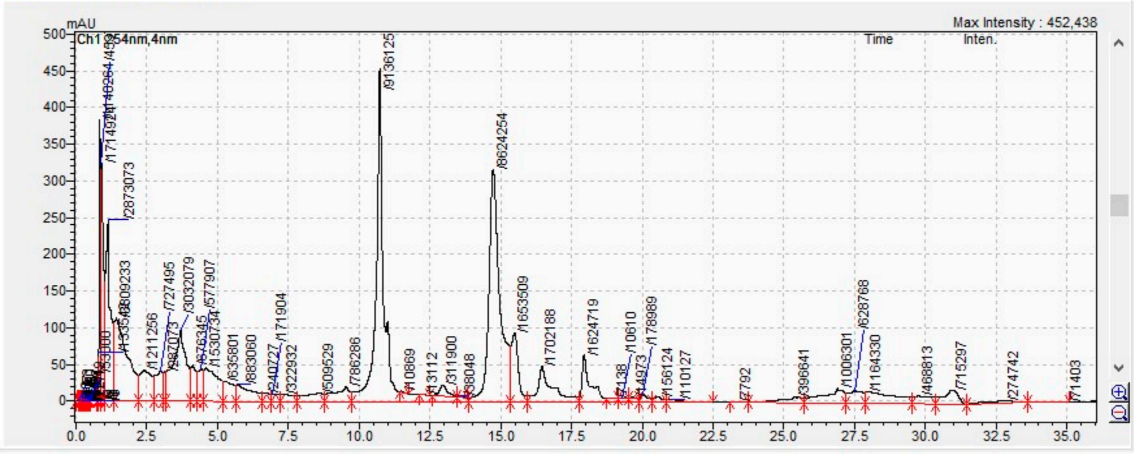

(**a**)

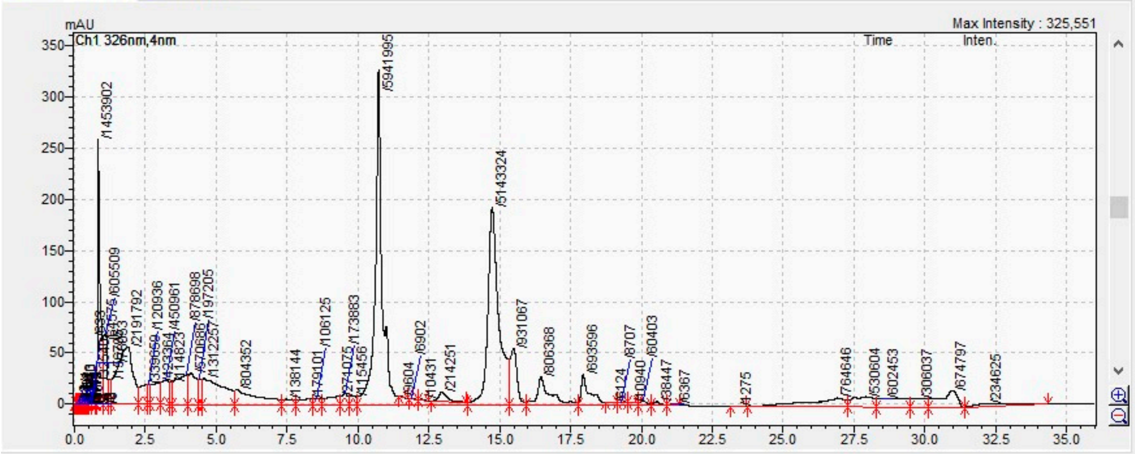

(**b**)

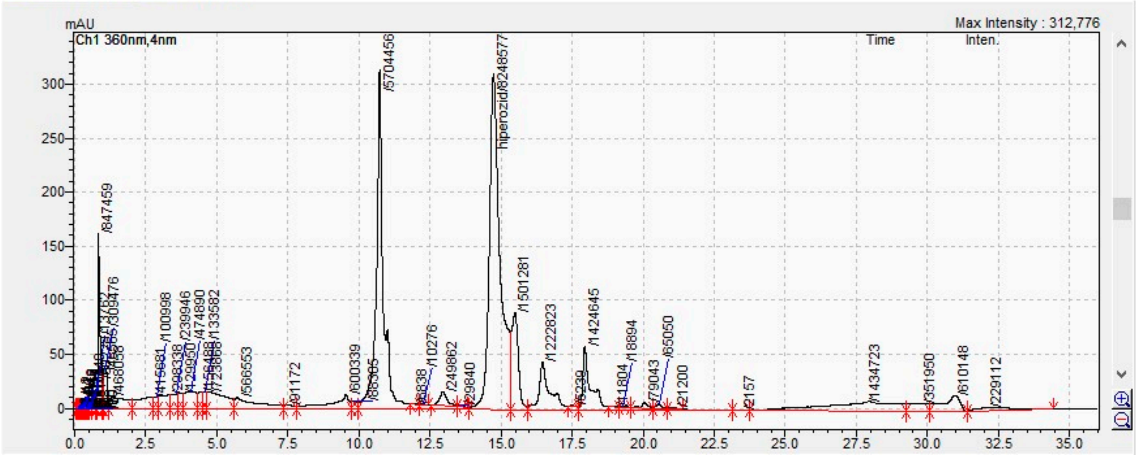

(**c**)

Figure S1. HPLC chromatogram of red onion dry extract at 254 nm, 326 nm and 360 nm. (a: 254 nm, b: 326 nm, c: 360 nm)

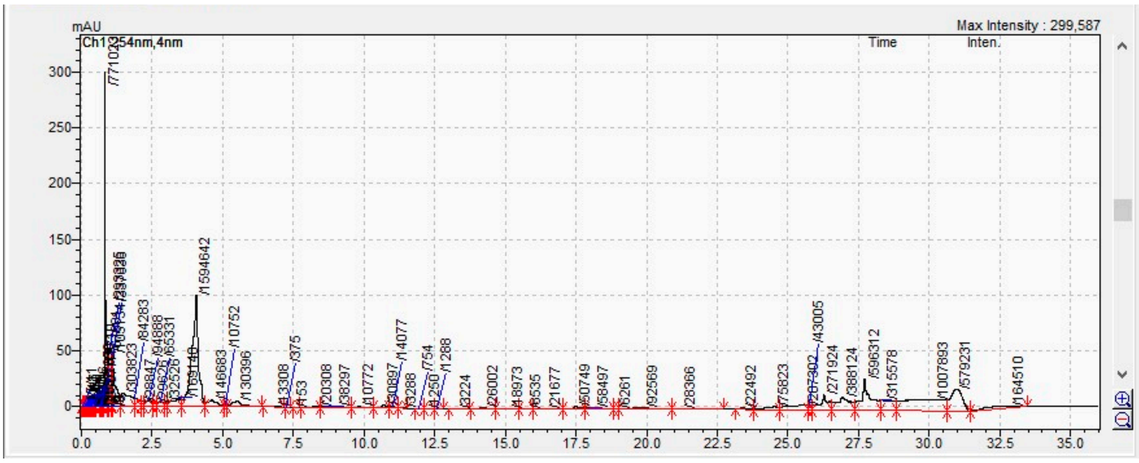

Figure S2. HPLC chromatogram of garlic dry extract at 254 nm.

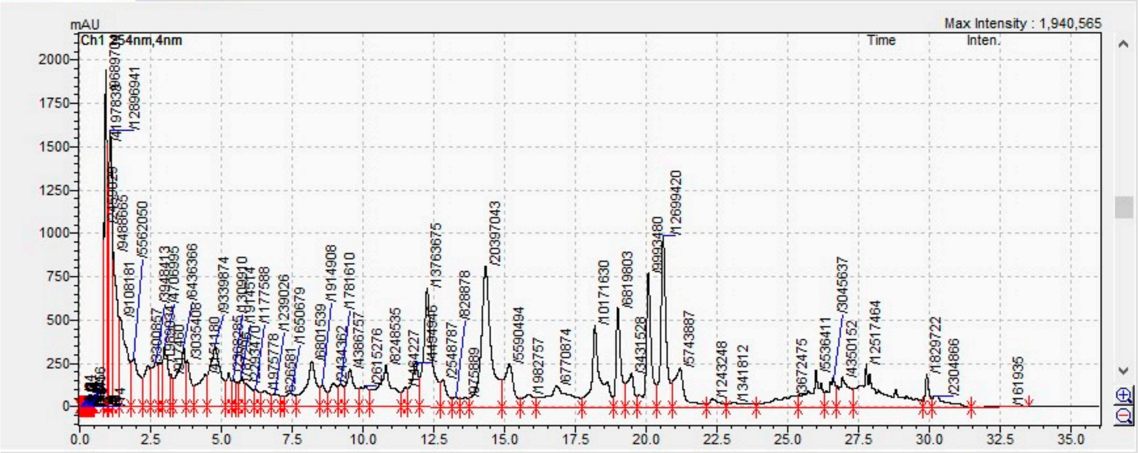

(**a**)

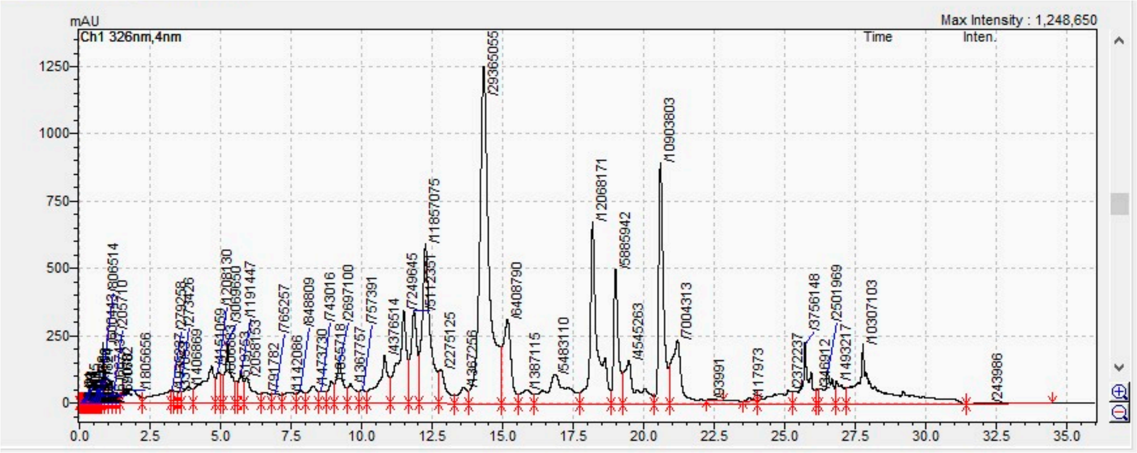

(**b**)

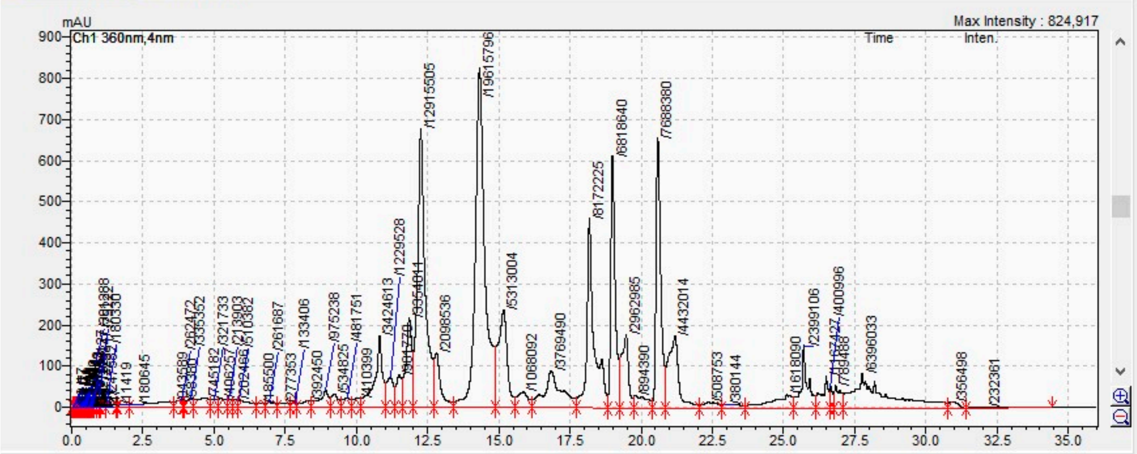

(**c**)

Figure S3. HPLC chromatogram of cumin dry extract at 254 nm at 326 nm and 360 nm. (a: 254 nm, b: 326 nm, c: 360 nm).

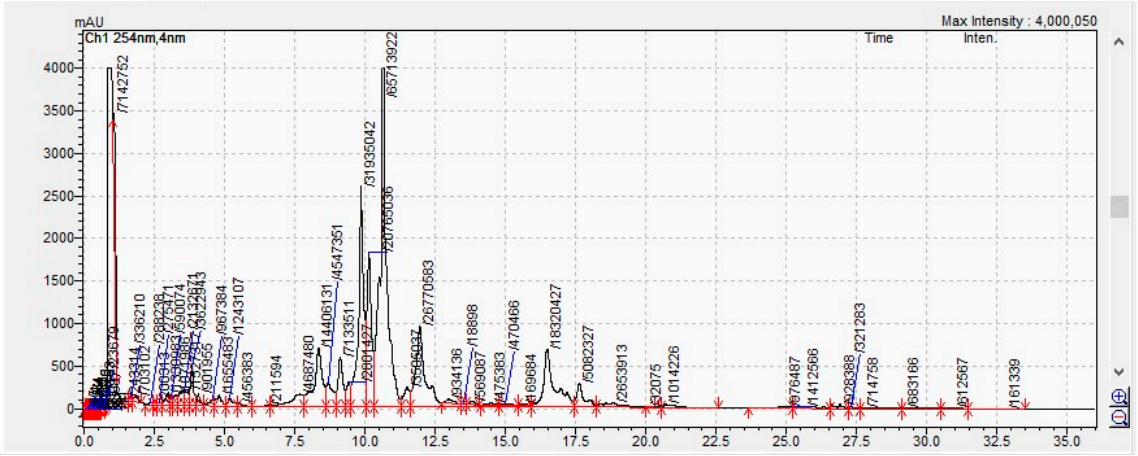

(**a**)

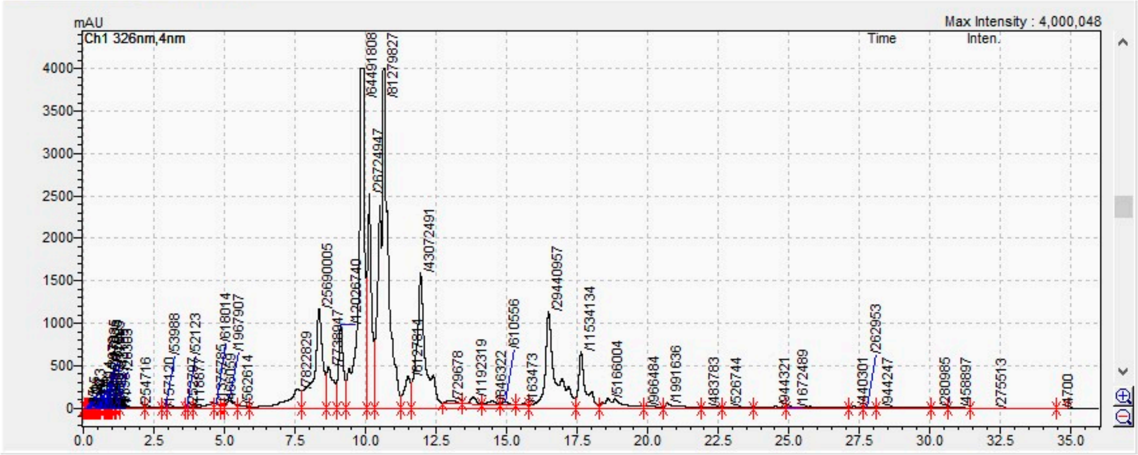

(**b**)

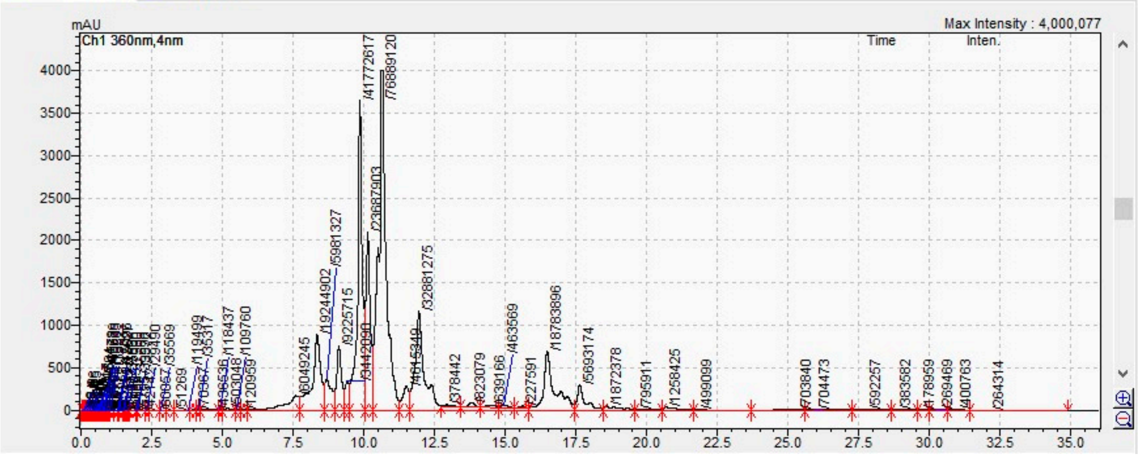

(**c**)

Figure S4. HPLC chromatogram of fenugreek dry extract at 254 nm, 326 nm and 360 nm**.** (a: 254 nm, b: 326 nm, c: 360 nm).

In the cumin extract, it is observed the separation of flavonoids at retention times between 10 and 20 min, but which are not derived from quercetin, having absorption maxima between 330 and 345 nm. At minute 20.030, a polyphenol appears that seems to be of the tannin class, probably a complex or condensed tannin. Tannins also appear at 4.864 min and 7.881 min, respectively. At retention times of more than 20 min, polyphenols appear that seems to be of the coumarin class. Cumin extract appears to be the richest in polyphenols. Among the flavonoids, those from 14.322 min and 12.280 min

respectively, that are the majority, representing 30.5% and 20.8%, respectively, of the total of the most important polyphenols. The rest are under 11%.

In the case of fenugreek extract at 3.115 min a tannin is separated, probably a gallic acid derivative, then at minutes 7.577, 10.146, 10.657 and 11.957 flavonoids with maximum absorption between 330 and 340 nm, and at over 15 minutes polyphenols that seems to be from coumarin class. At minute 9,838, a polyphenol with a spectrum specific to caffeic acid derivatives appears. Among the majority flavonoids, the predominant quantity is the one from minute 10.657, this being in proportion of 47.3%, and the component separated at 11.957 min in proportion of 26.9%.

Table S1. Polyphenolic compounds of plant extracts analyzed by HPLC.

Extract	Compound	Retention time (min)
Red onion	Gallic acid	3.137
	Unknown	3.687
	Quercitin	10.728
	Rutin	14.734
	Hyperoside	15.490
	Unknown	16.451
	Karempferol	17.967
Garlic	Gallic acid	5.904
Cumin	/	4.684
	/	7.881
	/	10.812
	Caffeic acid	12.280
	/	14.322
	Isoquercetine	15.165
	/	15. 857
	Vanillic acid	15.835
	/	18.179
	Syringaresinol	19.007
	/	19.487
	Myricetine 3-0 pentoside	20.030
	Citrusine	20.602
	Rosmarinic acid	21.197
	P-Coumaric acid	25.719
Fenugreek	Gallic acid	3.115
	Sinapic acid	7.577
	Caffeic acid	9.838
	Asterogenic acid	10.146
	/	10.657
	Pyrogallol	11.957
	Hyperoside	16.480
	Ferulic acid	17.647

/: Unidentified compound.
